# Oxidative stress in acute pulmonary embolism: emerging roles and therapeutic implications

**DOI:** 10.1186/s12959-023-00577-1

**Published:** 2024-01-12

**Authors:** Jingchao Yang, Jinzhu Xu, Shuanglan Xu, Zeqin Fan, Chenshao Zhu, Jianyuan Wan, Jiao Yang, Xiqian Xing

**Affiliations:** 1 Department of Pulmonary and Critical Care Medicine, First Affiliated Hospital of Kunming Medical University, 650032 Kunming, China; 2 Department of Pulmonary and Critical Care Medicine, Yuxi Municipal Hospital of T.C. M, 653100 Yuxi, China; 3 Department of Pulmonary and Critical Care Medicine, Affiliated Hospital of Yunnan University, 650021 Kunming, China

**Keywords:** Oxidative stress, Acute Pulmonary Embolism, Endothelial cells, Reactive oxygen species, Antioxidants

## Abstract

Oxidative stress is an imbalance between the body’s reactive oxygen species and antioxidant defense mechanisms. Oxidative stress is involved in the development of several cardiovascular diseases, such as pulmonary hypertension, atherosclerosis, and diabetes mellitus. A growing number of studies have suggested the potential role of oxidative stress in the pathogenesis of pulmonary embolism. Biomarkers of oxidative stress in pulmonary embolism have also been explored, such as matrix metalloproteinases, asymmetric dimethylarginine, and neutrophil/lymphocyte ratio. Here, we comprehensively summarize some oxidative stress mechanisms and biomarkers in the development of acute pulmonary embolism and summarize related treatments based on antioxidant stress to explore effective treatment strategies for acute pulmonary embolism.

## Introduction

Pulmonary thromboembolism (PTE) is a pathological process in which detached blood clots block the pulmonary artery or its branches. Impacted by anatomical obstruction and hypoxic vasoconstriction, pulmonary vascular resistance (PVR) and pulmonary artery pressure (PAP) increase if more than 30–50% of the total cross-sectional area of the pulmonary arterial bed is occluded by thromboemboli. The abrupt increase in PVR results in right ventricle dilation and right ventricular dysfunction. The dilation of the right ventricle leads to leftward bowing of the interventricular septum and a reduction in cardiac output, contributing to systemic hypotension and haemodynamic instability. Haemodynamic instability contains cardiac arrest, obstructive shock, or persistent hypotension, which delineates high-risk acute pulmonary embolism (APE). Accordingly, patients with stable haemodynamics are classified as low and intermediate risk. Evidence of right ventricle dysfunction or elevated cardiac troponin levels suggests intermediate early mortality risk [1].

According to the 2019 European Society of Cardiology (ESC) Guidelines for the diagnosis and management of acute pulmonary embolism, the initiation of anticoagulation is recommended without delay in patients with high or intermediate clinical probability of APE. All patients with APE should be treated with anticoagulants for at least 3 months. Low-molecular weight heparin (LMWH) or fondaparinux are often used among parenteral anticoagulants. Immediate anticoagulation and systematic thrombolytic therapy are recommended for high-risk APE. In addition, haemodynamic and respiratory support, vena cava filters, mechanical reperfusion, and surgical embolectomy are also ways to treat APE depending on the condition [1]. While anticoagulant treatment is effective in reducing recurrence, it is associated with a 1-2% annual risk of major bleeding [2].

Oxidative stress refers to an imbalance between reactive oxygen species (ROS) and antioxidant defense mechanisms in the body. A certain amount of ROS in mitochondria is now known to be biologically essential in a variety of physiological systems, including adaptation to hypoxia, autophagy, immunity, differentiation, and longevity [3], while excessive production of ROS leads to damage to lipids, proteins and deoxyribonucleic acid (DNA) [4–7]. In recent years, oxidative stress has been found in vascular diseases such as pulmonary hypertension (PH), atherosclerosis, and diabetes [8–10]. In addition, mitochondrial dysfunction, inflammation, and oxidative stress are also related to chronic thromboembolic pulmonary hypertension (CTEPH), as well as inoperable or residual CTEPH [11–14]. Similarly, many studies have found a profound link between oxidative stress and the onset and development of acute pulmonary embolism. A recent study revealed that PE led to increased ischemia-modified albumin (IMA) levels that were augmented following PE severity. Likewise, advanced oxidation protein products (AOPPs), proteins damaged by oxidative stress, may be used as clinical markers in the evaluation of APE severity in clinical practice [15]. Oxidative stress can alter mitochondrial metabolism and regulate pulmonary artery endothelial cell production and smooth muscle function through modulation of signal transduction pathways. Notably, oxidative stress related mechanisms and therapy are not introduced by the 2019 ESC guidelines for APE. This review provides an overview of possible oxidative stress mechanisms in the pathogenesis of APE and the potential drugs that are used to treat APE by targeting oxidative stress.

### Oxidative stress and antioxidant system

**Fig. 1:**
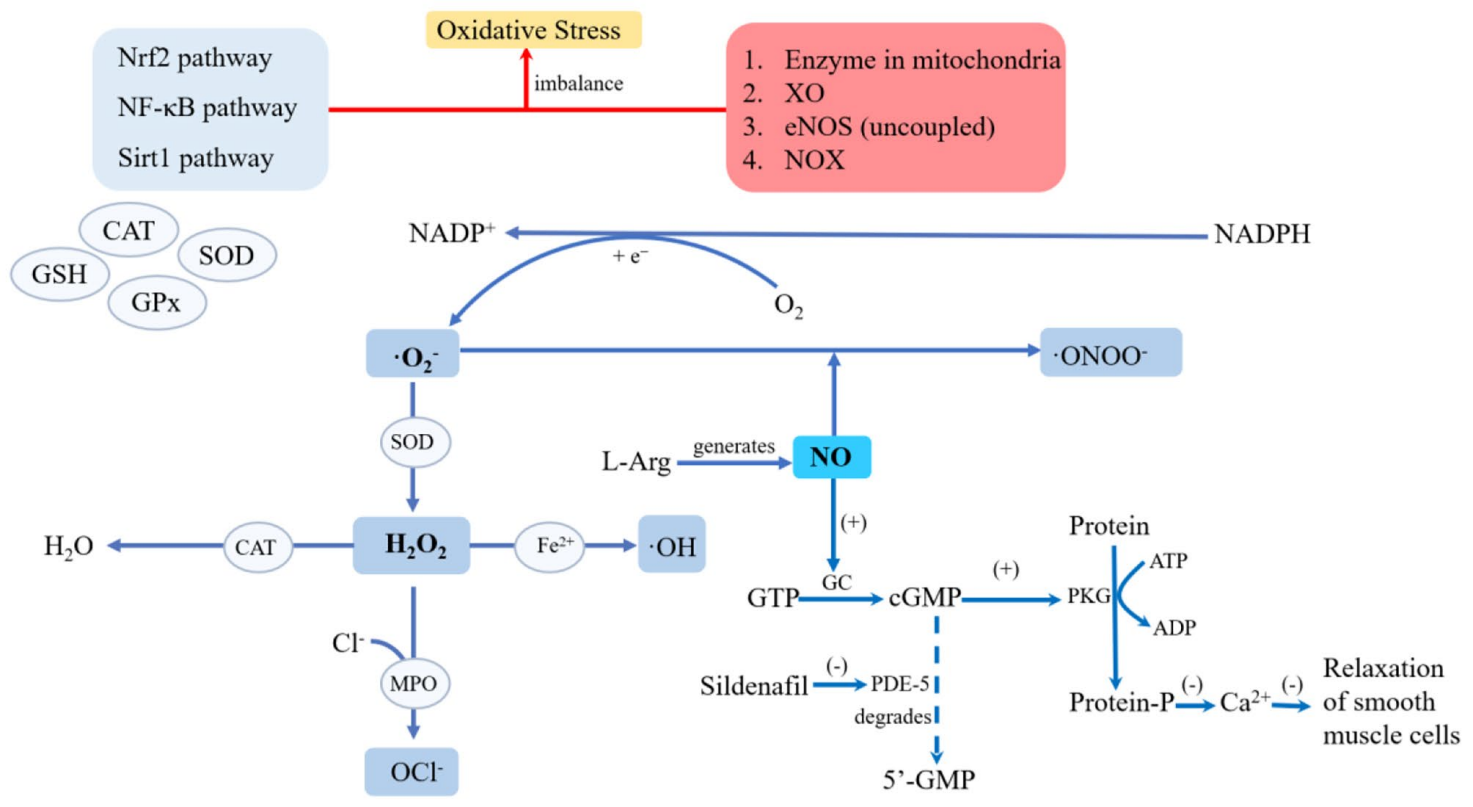
Metabolic pathways of ROS and how NO affects smooth muscle cells. Oxidative stress is the imbalance of the oxidative system and antioxidant system. Several enzymes and pathways form the antioxidant system. The ROS-producting system contains enzymes such as enzyme in mitochondria, XO, eNOS, and NOX. Under the catalysis of enzymes, O_2_^−^ and H_2_O_2_ are produced. ROS can either be transformed into water or strong oxidizing substances. NO relaxes smooth muscle by signal transduction. By inhibiting the cGMP pathway specifically, sildenafil promotes the relaxation of smooth muscle cells (ADP: adenosine diphosphate; ATP: adenosine triphosphate; CAT: catalase; cGMP: cyclic guanosine monophosphate; Cl^−^: chloride ion; eNOS: endothelial nitric oxide synthase; Fe2^+^: ferrous iron; GC: guanylate cyclase; GTP: guanosine triphosphate; H_2_O_2_: hydrogen peroxide; L-Arg: L-arginine; MPO: myeloperoxidase; NO: nitric oxide;NOX: nicotinamide adenine dinucleotide phosphate oxidase; NADPH: nicotinamide adenine dinucleotide phosphate-reduced; NADP^+^: nicotinamide adenine dinucleotide phosphate; NO: nitric oxide; O_2_: oxygen; ·O_2_^−^: superoxide;·ONOO^−^: peroxynitrite anion;·OH: hydroxyl radical; OCl^−^: hypochlorite ion; PDE-5: 5-phosphodiesterase; PKG: protein kinase G SOD: superoxide dismutase; XO: xanthine oxidase)

Superoxide (O_2_^−^) and hydrogen peroxide (H_2_O_2_) are the main ROS involved in the regulation of cellular metabolism [16]. When moleculer oxygen acquires one electron, O_2_^−^ comes into being. The process is executed by ROS-producing systems that primarily contain enzymes involved in the mitochondrial respiratory chain, including nicotinamide adenine dinucleotide phosphate oxidase (NOX), uncoupled nitric oxide synthase (NOS), and xanthine oxidase (XO) [17]. The NOX1 and NOX2 oxidases are the major sources of ROS in the artery wall in hypercholesterolaemia and contribute to oxidative stress and endothelial dysfunction [18]. In experimental mice with APE, inhibition of NOXs increased intracellular cyclic guanosine monophosphate (cGMP) for antithrombosis [19]. XO is an enzyme that catalyses the conversion of hypoxanthine to xanthine and then xanthine to uric acid, mediating the generation of ROS [20]. Endothelial NOS (eNOS) is an important enzyme for the production of nitric oxide (NO), and eNOS levels were significantly decreased in APE model rats, accompanied by a significant decrease in the expression of sirtuin-2 (SIRT2) and an increase in the expression of nuclear factor κB (NF-κB), a redox-sensitive nuclear transcription factor, which is highly associated with ROS generation and controls the expression of antioxidant genes in the vasculature. The nuclear factor erythroid-2-related factor 2 (Nrf2) pathway is vital in clearing and degrading accumulated ROS by regulating the expression of antioxidant defense genes in the vasculature [21]. In the state of oxidative stress, enzymatic production of ROS exceeds the available antioxidant defense systems. The metabolic pathways of reactive oxygen species are listed in Fig. 1, and the cited reference demonstrated the oxidative stress process in vascular disease [22] (Fig. 1).

To detoxify and neutralize accumulated ROS, vascular endothelial cells are equipped with an antioxidant defense system involving antioxidant enzymes, such as superoxide dismutase (SOD), catalase (CAT), glutathione peroxidase (GPx), and nonenzymatic antioxidant compounds, such as glutathione (GSH), to maintain a steady intracellular redox state [17].

### Role of NO in oxidative stress in PTE

NO has a dual role under physiological and pathophysiological conditions. An appropriate amount of NO can regulate endothelial cell function and inhibit the proliferation of smooth muscle cells, and anti-platelet and anti-inflammatory factors. However, excessive NO is converted into peroxynitrite (ONOO^−^), which damages vascular endothelial cells [23–27]. eNOS is distributed in the endothelium of large pulmonary vessels [28]. A functional eNOS oxidizes its substrate L-arginine to L-citrulline and NO·. It is the predominant NOS isoform in the vasculature and is responsible for most of the NO· produced in cardiac tissue. Vascular NO· dilates all types of blood vessels by stimulating soluble guanylyl cyclase and increasing cGMP in smooth muscle cells [29]. Moreover, NO· released in the vascular lumen is a potent inhibitor of platelet aggregation and adhesion. NO· can also inhibit leukocyte adhesion to the vessel wall either by interfering with the ability of the leukocyte adhesion molecule cluster of differentiation 11 (CD11)/CD18 to form an adhesive bond with the endothelial cell surface or by suppressing CD11/CD18 expression on leukocytes [29]. And the significance of platelets and leukocytes is summarized in the *mechanism* section. Decreased or no expression of eNOS was observed in patients with PH, and a reduction of NOS may lead to pulmonary vasoconstriction and excessive growth of the tunica media in PH [30]. The inducible NOS (iNOS) isoform may play a key role in the oxidative stress of APE. Although the iNOS inhibitor S-methylisothiourea reduced plasma lipid peroxide levels, it could neither reverse APE-induced PH nor synergistically improve hemodynamics in combination with sildenafil [31]. As a PDE-5 inhibitor, sildenafil has been widely used in pulmonary artery hypertension, which participates in the NO-sGC (soluble guanylate cyclase)-cGMP pathway. The effect of sildenafil is pulmonary vasodilation. The pathway is shown in Fig. 1 [32]. Interestingly, red blood cell eNOS directly contributes to systemic NO bioavailability and blood pressure homeostasis, independent of vascular endothelial eNOS [33]. L-arginine can resist APE-induced PH by promoting the NO pathway. And this process is reversed by the NOS inhibitor, hydrochloride [34]. In a rabbit model of massive pulmonary embolism, sympathetic medium transmitters tyrosine hydroxylase and neuropeptide Y were upregulated in whole lung tissues, and intravenous sodium nitroprusside reduced mean pulmonary arterial pressure (mPAP) and treated cardiogenic shock by relieving vasospasms in both pulmonary embolism and non-pulmonary embolism areas. Sodium nitroprusside lowers the levels of sympathetic medium transmitters and increases levels of NO [23]. These studies demonstrate that NO plays an essential role in the regulation of vascular homeostasis. (Fig. 1)

APE is a disease associated with intravascular haemolysis, which produces free plasma haemoglobin. Treatment with a guanylate cyclase stimulator normalized pulmonary hemodynamics, reduced hemolysis, and improved oxygenation [35]. Early research suggested that the release of haemoglobin during intravascular haemolysis resulted in excessive consumption of NO, which subsequently led to decreased activity of guanylate cyclase, contraction of smooth muscle, vasoconstriction, and platelet activation/aggregation. Many of the proinflammatory effects of plasma haemoglobin may involve the consumption of NO [36]. In an animal model of APE, NO consumption was proportionally increased with the plasma haemoglobin concentration, while the free radical scavenger Tempol alleviated APE-induced haemolysis, reduced NO consumption and improved APE-induced hemodynamic disorders and PH [37, 38]. Consequently, haemolysis may be part of the oxidative stress mechanism in APE.

### Mechanisms of oxidative stress associated with APE

PE results in pulmonary vascular hypertension by pulmonary artery vasoconstriction and mechanical obstruction of the vasculature with clot material [35]. Contraction of pulmonary artery smooth muscle cells (PASMCs) leads to pulmonary artery constriction, and mechanical obstruction causes abnormal perfusion of the lung.

#### Hypoxia of pulmonary smooth muscle cells

Whereas most systemic arteries dilate in response to hypoxia, those in the pulmonary circulation constrict, which is known as hypoxic pulmonary vasoconstriction. Hypoxic pulmonary vasoconstriction is an adaptive mechanism that during localized alveolar hypoxia diverts blood away from poorly ventilated regions of the lung, thereby preserving ventilation/perfusion matching [39]. Acute PE increases pulmonary vascular resistance, partly attributable to hypoxic vasoconstriction [40]. The hypoxia-induced increase in Ca^2+^ concentration in PASMCs promotes PASMC contraction, thereby triggering pulmonary vasoconstriction [41]. It has been reported that the rotenone binding site nicotinamide adenine dinucleotide dehydrogenase ubiquinone iron-sulfur protein 2 of mitochondrial complex I is a redox-sensitive pulmonary vascular oxygen sensor. Hypoxia inhibits complex I in smooth muscle cells and elicits pulmonary vasoconstriction [42]. The contraction of PASMCs under hypoxic conditions requires mitochondrial ROS. Mitochondria-targeted antioxidants can block hypoxic pulmonary vasoconstriction in isolated rat lungs under acute hypoxia conditions. During acute hypoxia, mitochondrial complex III in smooth muscle cells releases superoxide into the cytoplasm, increasing the concentration of cytoplasmic Ca^2+^ and thereby inducing acute hypoxic pulmonary vasoconstriction [43, 44].

#### Ischemia-reperfusion, absence of blood flow in shear stress, and activation of leukocytes

The pathophysiological changes of APE are manifested by increased pulmonary vascular resistance and cardiac and respiratory insufficiency. Mechanical obstruction of the pulmonary artery may play a very important role in APE. Among the many mechanisms of APE, there are at least three mechanisms that are related to oxidative stress: ischemia-reperfusion, changes in blood flow in shear stress, and activation of leukocytes. These mechanisms are not independent and may be secondary to mechanical obstruction of the pulmonary artery.

Pulmonary embolism leads to the change or interruption of pulmonary artery blood flow. Physiologically, steady laminar shear stress and pulsatile flow reduce the levels of ROS generated in the endothelial cell matrix and mitochondria, and maintain posttranslational oxidative modifications of proteins that are needed for endothelial cell physiology, whereas during ischemia and reperfusion, high levels of ROS are generated in endothelial cells that lead to altered mitochondrial dynamics and endothelial cell apoptosis [45–47]. Laminar shear stress promotes transient H_2_O_2_ production and activates the p38 MAP kinase pathway, which activates eNOS to produce NO and protects endothelial function [48]. A study by Chatterjee et al. provided evidence that the cessation of blood flow during lung ischemia is *sensed* by the endothelium via a mechanosignaling cascade that depolarizes endothelial cell membranes and activates the phagocytic isoform of NOX2 to generate ROS and NO. This process ultimately leads to oxidative injury and activation of signaing pathways that drive inflammation and cell death. It was reported that the earliest shear sensing event occurred in a K^+^ channel. A K^+^-adenosine triphosphate (ATP) channel in the lung endothelium is responsible for maintaining membrane potential with normal shear stress and is inactivated by the its loss, leading to endothelial membrane depolarization, which is a key component in the cell signalling cascade. The mechanosensor recognizes mechanical forces and converts mechanical stimuli into biochemical reactions that can induce mechano-dependent cellular responses [49]. Flow-adapted endothelial cells respond to cessation of flow with increased ROS production, resulting in activation of NF-κB and activator protein-1 (AP-1) and cellular proliferation [50]. Reperfusion led to further oxidant production than ischemia, and increased ROS production with reperfusion was NOX2 dependent [45].

Liu Z et al. proposed that endothelial cell damage is the first element of pulmonary embolism [51]. After that, the hyperactivation of leukocytes was discovered. Endothelial cells act on platelets and neutrophils through biomolecules such as microparticles and tissue factors to stimulate thrombosis. Earlier studies have shown that endothelial cells, leukocytes, and platelets are significantly activated in venous thromboembolism (VTE) patients, accompanied by the release of endothelial microparticles (EMPs) and the formation of EMP-monocyte conjugates and platelet-leukocyte conjugates [52]. Endothelial cell dysfunction is observed after an acute PTE episode and manifests as low e-selectin and high soluble intercellular cell adhesion molecule-1 (sICAM-1). ICAM causes leukocytes to adhere to the endothelial cell surface firmly and migrate across the endothelium, whereas E-selectin regulates the binding of leukocytes to damaged endothelial cells. Both sICAM-1 and E-selectin were found to be sensitive markers of endothelial cell dysfunction [53]. Peripheral blood leukocyte count is a widely used indicator of the inflammatory response. Early studies demonstrated the presence of inflammatory responses in lung tissue by elevated platelet-activating factor (PAF) and neutrophil numbers in the bronchoalveolar lavage fluid of patients with PTE [54]. Four hours after reperfusion, the pulmonary microvascular permeability reached a second peak, which was associated with the production of ROS by neutrophils. [55]. A study by microarray technology examined the gene expression profile after lung ischemia-reperfusion injury and found that 169 genes were significantly altered. Many of these genes are associated with inflammatory responses, such as matrix metalloproteinases (MMP-8, MMP-9), chemokines (S100a8, S100a9, and Fpr1) and inflammatory cytokine receptors (receptors of interleukins: IL-1R2 and IL-8Rb) [56]. Chemokine increases in the right ventricle of rats with APE, such as cytokine-induced neutrophil chemoattractant-1 (CINC-1), CINC-2, monocyte chemotactic protein-1 (MCP-1), and macrophage inflammatory protein (MIP-1). Subsequently, neutrophils and monocytes accumulate in the right ventricle. In this process, the levels of IL-1, IL-6, and e-selectin also increase sharply [57, 58]. Anti-CINC-1 treatment inhibited the accumulation of neutrophils in the right ventricle of APE model rats, and plasma troponin I concentrations also decreased [59].

Another important aspect concerning leukocyte overactivation in APE is neutrophil extracellular traps (NETs). Recently, lysophosphatidic acid (released by activated platelets) in APE patients has been found to activate neutrophils to release NETs, generate anti-tPA effects on blood clots, and promote thrombotic stability [60]. Citrullinated histone H3, a specific marker of NETs, was found to be significantly increased in APE patients. However, its accuracy in the diagnosis of VTE is not higher than that of D-dimer [61, 62].

**Fig. 2:**
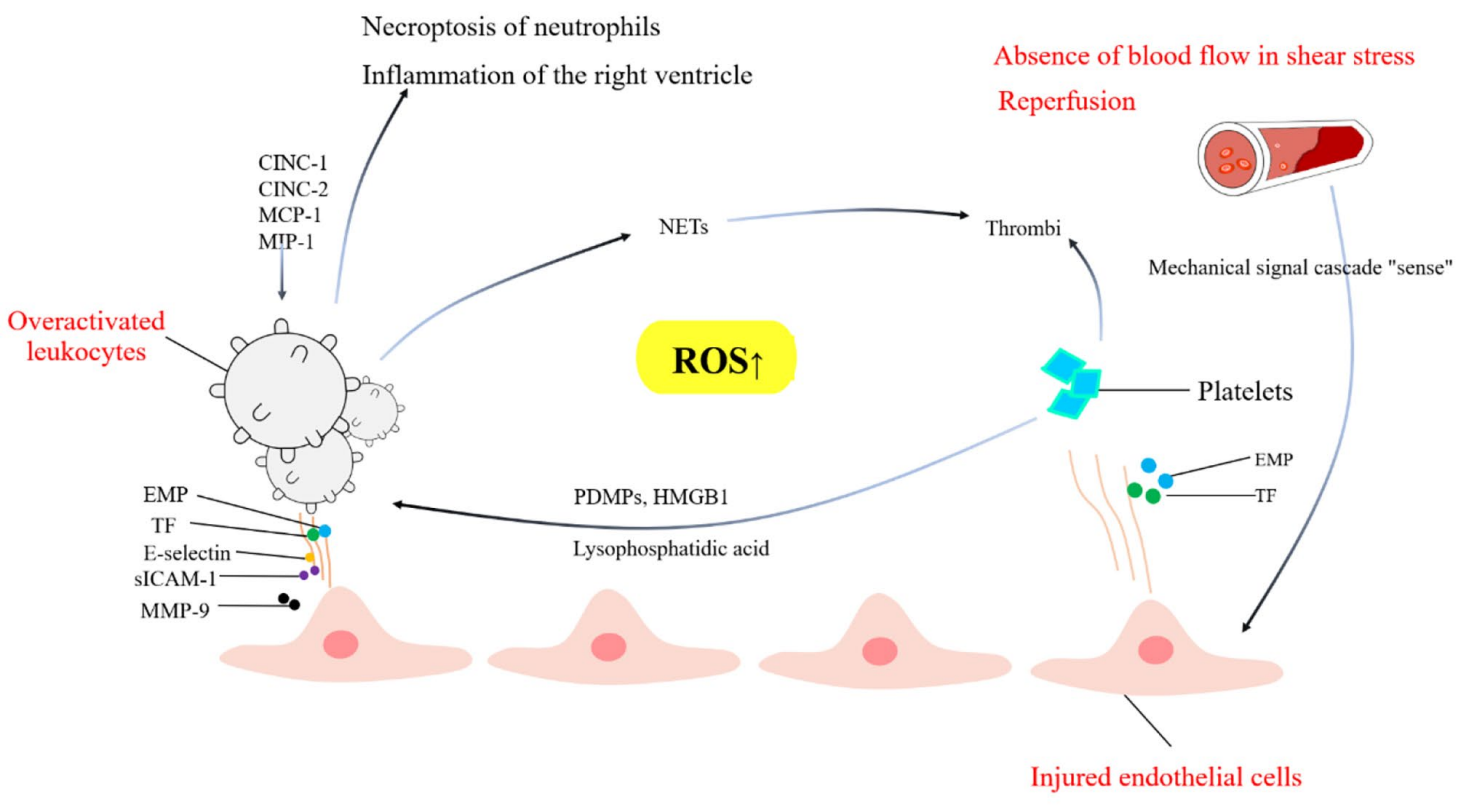
Partial mechanisms of APE (red font). Under conditions of oxidative stress, endothelial cells become injured, and tissue factors and endothelial microparticles are released to activate leukocytes and platelets. sICAM and E-selectin were abnormally elevated. Meanwhile, leukocytes are overactivated by CINC-1, CINC-2, MCP-1, and MIP-1. Lysophosphatidic acid released by activated platelets stimulates neutrophils to release neutrophil extracellular traps, which promote thrombotic stability. High mobility group box 1 and platelet-derived microparticles also activate neutrophils to trigger venous thrombosis. Besides, the absence of blood flow in shear stress and reperfusion causes damage to endothelial cells via a mechanical signal cascade “sense”. These mechanisms interact to aggravate oxidative stress in APE (APE: acute pulmonary embolism; CINC: cytokine-induced neutrophil chemoattractant; EMP: endothelial microparticle; HMGB: high mobility group box 1; sICAM: soluble intercellular cell adhesion molecule; MCP: monocyte chemotactic protein; MIP: macrophage inflammatory protein; MMP-9: matrix metalloprotein-9; NETs: neutrophil extracellular traps; PDMP: platelet-derived microparticles; TF: tissue factor)

Venous thrombotic disease is associated with interactions between platelets and neutrophils. The death of neutrophils in VTE is partially due to the activation of platelets [63]. Activated platelets and platelet-derived microparticles (PDMPs) act on p-selectin to activate leukocytes [64, 65]. High mobility group box 1 (HMGB1), a characteristic protein released after activation of innate immunity, was found to be elevated in abnormal coagulation states. Then platelet-derived HMGB1 recruits neutrophils to promote neutrophil aggregation and activates NET generation to trigger venous thrombosis [66]. In acute intermediate-risk PE patients, platelets showed a > 30% increase in mitochondrial oxygen consumption, and mitochondrial ROS production [67]. Early cases suggested that platelet-p-selectin, PDMP, chemokines MCP-1 and soluble E-selectin, soluble vascular cell adhesion molecule-1 (sVCAM-1), and soluble CD14 were significantly and abnormally elevated in PE patients (Fig. 2) [68].

### ROS-related biomarkers in APE

Evidence regarding oxidative stress biomarkers in patients or animal models with APE is separately reviewed in the following paragraphs.

#### MMP-9

Matrix metalloproteinases (MMPs) include a group of more than 20 zinc-dependent enzymes involved in remodeling several components of the extracellular matrix. During an inflammatory response, leukocyte traffic through tissue barriers, including basement membranes, is only possible if these cells are equipped with enzymes that can remodel the ECM. MMPs are therefore crucial effector molecules of inflammatory cells [69]. Increased levels of MMP-9 are closely associated with APE, while increased MMP-2 levels are not found in the right ventricle of APE patients [70, 71]. Doxycycline, a nonspecific MMP inhibitor, inhibited MMP-9-related ventricular myocardial protein degradation and myocardial ROS production in APE, alleviated APE-induced right ventricular injury, prevented right ventricular thinning and chamber enlargement to some extent, and reduced mortality in rats [71, 72]. Tempol is an ROS scavenger that reduces ROS and MMP-9 levels in the right ventricle of APE models. In addition, it prophylactically and therapeutically lowers mPAP and the pulmonary vascular resistance index [70, 73]. Sildenafil is a specific 5-phosphodiesterase inhibitor of the cGMP pathway that attenuates APE-induced lipid peroxidation damage. Sildenafil improves hemodynamics and reduces MMP-2 and MMP-9 activity in a rabbit model of acute PTE, and the combination of N-acetylcysteine enhances the action of sildenafil by reducing neutrophil exudation and 8-isoprostane consumption [74, 75]. Atorvastatin attenuates APE-induced oxidative stress and protein leakage in the alveoli, inhibits MMP activation, and prevents PH associated with APE, with or without sildenafil [76]. Certain concentrations of nitrite combined with sildenafil reversed APE-induced PH, reduced pulmonary vascular resistance, inhibited MMP-9 and the levels of elevated oxidative stress marker thiobarbituric acid, whereas the nonantioxidant BAY41-2272 stimulated only the NO-cGMP pathway and had an effect on hemodynamics, without affecting MMP and oxidative stress levels, which corroborates the association of MMP with oxidative stress [77]. As mentioned before, tissue damage in APE is associated with ROS and MMPs secreted by neutrophils, and early studies found that ROS activate MMP-9 and that the two synergize with each other to exacerbate this tissue damage [78].

#### ADMA

Asymmetric dimethylarginine (ADMA) is present in a variety of tissues and is associated with endothelial cell dysfunction and multiple chronic diseases. Intracellularly, L-arginine is degraded by protein arginine methyltransferases (PRMTs) and is hydrolysed to produce ADMA, which in turn is degraded by dimethylarginine dimethylamine hydrolase (DDAH) to citrulline. ADMA can reduce NO production by inhibiting NOS, leading to endothelial cell injury [79–82]. In a follow-up study of PTE, ADMA levels were significantly higher in patients than in the healthy population and they did not significantly decrease after 1 month of treatment. In addition, IL-6 levels in the PTE patients were significantly higher than those in the control group, suggesting that ADMA may be involved in inflammatory and oxidative stress processes and confirming the persistence of an inflammatory response in PTE patients for a while after treatment [83]. However, there are conflicting studies regarding the relationship between deep vein thrombosis and ADMA. Some studies have found a lack of association between ADMA and deep vein thrombosis [84, 85].

#### HSP90

Heat shock protein (HSP) is a highly conserved protein in living organisms and plays a role in cell protection during organismal stress. A case-control study showed that serum HPS27 and HSP90 concentrations were significantly higher in patients with APE than in healthy controls and were negatively correlated with arterial partial pressure of oxygen/arterial oxygen saturation (PaO_2_/SaO_2)_, suggesting that HSP27 and HSP90 may be associated with hypoxia in APE [86]. When human umbilical vein vascular endothelial cells were incubated with plasma from acute PTE patients, HSP90 mRNA was significantly elevated, which increased NO release by activating eNOS, and HSP90 expression was present in PTE patients within 30 min. Moreover, the HSP90 inhibitor geldanamycin suppressed the increase in cGMP, and NO synthesis was inhibited. This finding suggests a direct link between HSP90 and eNOS activation [87].

#### Hcy

Homocysteine (Hcy) is an intermediate product of methionine metabolism and is an independent risk factor for cardiovascular system disease. In recent years, Hcy levels have been found to be higher in patients with pulmonary embolism than in normal subjects and are a risk factor for the development of VTE [88–90]. High levels of Hcy induce endothelial cell apoptosis and involve mitochondrial activation. In addition, high levels of Hcy inhibit cytochrome oxidase (COX) activity, decrease COX17 expression, increase intracellular ROS levels, and induce endothelial cell apoptosis. Folic acid can attenuate the above effects of Hcy [91]. It may be important to monitor blood Hcy levels to prevent pulmonary embolism.

#### GGT

Gamma glutamyl transferase (GGT) is an enzyme synthesized primarily by the liver and is involved in the extracellular catabolism of glutathione. GGT plays a prominent role in the homeostasis of glutathione production and recycling, inflammatory and NO signaling, and oxidative stress amelioration. GGT levels increase with the number of pulmonary artery segments involved in APE and are significantly correlated [92]. Elevated GGT is associated with hemodynamic disturbances (hypotension and tachycardia) in patients with APE. When the cutoff value is specified as > 55 IU/L, GGT is sensitive in predicting the risk of early death from APE. However, the pathogenic relationship between GGT and APE is not yet clear [93]. A long-term, retrospective study with a large sample size showed that GGT and the neutrophil/lymphocyte ratio (NLR) are reliable prognostic biomarkers during the follow-up of PH and CTEPH. Their predictive power is comparable to the current gold standard for risk stratification [94]. However, GGT distributed in multiple organs, and there is an inherent lack of specificity in GGT [95].

#### NLR

In a prospective multicentre study, neutrophil/lymphocyte ratio levels were significantly higher in patients with APE than in patients without APE. Moreover, the NLR was strongly associated with mortality prognosis in low- and intermediate-risk APE patients but not in high- or intermediate-high-risk APE patients. [96]. The study combined NLR with rapid field assessment metrics for APE (cardiac troponin I, cTnI and N-terminal pro-brain natriuretic peptide, NT-proBNP) to create a right ventricular dysfunction risk score formula to predict right ventricular dysfunction and 30-day mortality in patients with APE more accurately [97].

#### IMA, AOPPs and FRAP

Ischemia-modified albumin (IMA) is an altered type of serum albumin that forms under conditions of oxidative stress. IMA is associated with a variety of diseases, such as acute coronary syndrome, skeletal muscle ischemia, and acute mesenteric ischemia. [98–100]. Studies by Suleyman and Turedi et al. showed that IMA levels were significantly higher in PE patients than in healthy individuals. Furthermore, IMA levels may be useful for the diagnosis of PE as a discriminative marker to exclude PE [101, 102]. However, larger studies are still needed. Gülseren et al. found that IMA could be used to evaluate the severity of APE. In the study, the levels of AOPPs were also significantly higher in patients with high-risk PE than in the intermediate and low-risk groups. Another indicator in the study, prooxidants-antioxidants balance (PAB), did not differ statistically significantly between the high-risk and intermediate-risk or low-risk groups. The fourth indicator of this study, ferric reducing antioxidant power (FRAP), had significantly higher levels in APE patients than in the control group, but there was no significant difference in the low-risk and intermediate-risk groups [15]. The indicators above may have vital potential for the diagnosis and risk stratification of APE.

### The anti-inflammatory effect of anticoagulants

As mentioned in the “introduction” part, treatment strategies differ depending on risk classifications. Anticoagulation and reperfusion treatment are necessary for high-risk APE patients. Until the haemodynamics stabilize, parenteral anticoagulation can be switched to oral drugs. The transition time point should be based on clinical judgement. For intermediate-risk APE, parenteral or oral anticoagulation is an adequate treatment, and routine primary reperfusion treatment is not recommended because of the high risk of fatal bleeding. Low-risk patients with APE should receive anticoagulation treatment in the hospital or early discharge and home treatment. Previous research has suggested that some commonly used anticoagulants prevent inflammatory damage to orgrans.

#### Heparin and its derivatives

It is effective to use heparin in inflammatory conditions such as acute coronary syndrome, cardiopulmonary bypass, and thrombophlebitis. Theoretically, heparin is able to interfere with several stages of leukocyte transmigration and extravasation into the target tissue [103]. Enoxaparin is one of the LMWHs, and its cardioprotective effect against doxorubicin is proven via suppression of oxidative stress, inflammation and apoptosis [104]. In rabbits with APE, LMWH is able to inhibit the local inflammatory reaction, which manifests as a decrease of IL-13 levels [105].

#### DOACs

In fact, direct oral anticoagulants (DOACs), such as rivaroxaban, dabigatran, apixaban, and edoxaban, not only exert anticoagulation effect, but also fight inflammation. To conclude, they are pleiotropic. There is a review about the effects of DOACs in endothelial cells in vitro. Potential differences between the four agents are also discussed [106]. In a cardiac injury rat model, rivaroxaban decreases cardiac oxidative stress, inflammation, and platelet reactivity [107]. A study performed on human umbilical vascular endothelial cells has shown that both rivaroxaban and dabigatran can induce indirect repair of DNA by inhibiting ROS production. Moreover, dabigatran may have a stronger antioxidant activity than rivaroxaban [108]. Anti-inflammatory and antioxidant effects of rivaroxaban have also been discovered in patients with APE [109].

### Antioxidant treatments for APE

The antioxidant drugs listed below are not yet recommended by the guidelines, however, emerging studies have shown their therapeutic roles in APE. The following western drugs and herbal extracts have been proven to be effective for the treatment of APE by targeting oxidative stress.

#### Statins

Atorvastatin and simvastatin inhibited APE-induced PH, which was related to antioxidative stress effects. NF-κB expression was inhibited in simvastatin-treated APE model rats by increased expression of SIRT2 and eNOS. This inhibition affected the levels of downstream inflammatory factors, resulting in a significant reduction in pulmonary artery wall and tissue inflammation and a decrease in pulmonary vascular resistance, mPAP, and right ventricular load. Simvastatin improves acute PTE-induced PH and unstable hemodynamics, most likely by regulating the SIRT2/NF-κB signalling pathway [21]. Similarly, atorvastatin attenuated PH, improved the 24-hour survival rate, and reduced MMP-9 levels in APE model rats [110]. Statins alleviate the inflammatory response in blood vessels by inhibiting adhesion molecules [111]. A meta-analysis showed that statins may also reduce the recurrence of VTE [112].

#### NO related therapy

Inhalation of NO alone does not significantly affect platelet mitochondrial oxygen consumption or platelet function in patients with submassive PE. NO inhibits platelet aggregation in vitro via the sGC/cGMP/PKG pathway. Moreover, platelet soluble guanylate cyclase (sGC) is inactivated by oxidation during PE and loses its response to NO. The sGC activator cinaciguat reduces platelet intracytoplasmic Ca^2+^ concentrations, whereas the sGC stimulator riociguat does not [113]. In an intermediate-risk APE model in pigs, both NO or riociguat inhalation reduced pulmonary vascular resistance without lowering systemic blood pressure and improved hemodynamics to some extent. The former was achieved via the NO-sGC-cGMP pathway [114]. Inhaled NO in patients with intermediate- to high-risk pulmonary embolism failed to maintain troponin T, BNP, and right ventricle function at normal levels but was able to inhibit right ventricular hypokinesis and ventricular dilation [115]. Nilsson, K. F. et al. synthesized a novel organic nitrite, 1,2-propanediol, which can act as an NO donor and significantly reduce the APE-induced increase in pulmonary vascular resistance without causing tolerance or significant side effects [116].

#### Apelin/APJ

The apelin receptor (APJ), separated by O’Dowd et al., is a member of the seven-transmembrane G protein-coupled receptor family. Apelin is an endogenous ligand of APJ, and its C-terminus is the specific binding region of the APJ receptor. In dogs with APE, vasoconstriction caused by vasoactive substances can be reversed by apelin-mediated NO production. Indeed, the apelin-induced vasodilation effect is associated with NO-dependent mechanisms. However, apelin-induced phosphorylation of myosin light chains in vascular smooth muscle cells can promote vasoconstriction [117]. Currrently, the function of apelin in APE is poorly clarified, and more exploration is needed.

#### GSK669

NOD2 is a pattern recognition receptor critical for innate immunity and inflammation, while GSK669 is a specific NOD2 receptor antagonist. Pan et al. found the GSK669 alleviates oxidative stress in a pulmonary embolism model as a glycoprotein VI antagonist and inhibits ROS generation from platelets upon glycoprotein VI activation. APE animal model experiments demonstrated that GSK669 not only inhibited the release of inflammatory substances and platelet aggregation from mobile platelets but also inhibited collagen-related peptide-induced ROS production, significantly reduced malondialdehyde (MDA, an indicator that reflects intracellular oxidative stress) levels, and increased SOD levels in plasma [118].

#### Flavonoids

Flavonoids play a commendable role in reducing oxidative stress in type-2 diabetes mellitus, dinitrobenzene sulfonic acid-induced colitis and Alzheimer’s disease [119]. Additionally, they are regarded as antioxidants that reduce ROS levels and thus inhibit platelet function [120]. A high dose of baicalin, which is a natural flavonoid, can reduce mPAP and right ventricular pressure levels and improve the symptoms of PH in APE model rats; a certain dose of baicalin can correct the hypoxic state of the organism. Baicalin induced the upregulation of p-IκBα protein expression levels and the downregulation of p65 protein expression in the NF-κB signalling pathway [121]. Astragalin, a flavonoid, is suggested to block thrombin-induced protease-activated receptor (PAR) signalling leading to alveolar inflammation. Besides, it inhibits ROS-mediated activation of MAPK signalling pathways in thrombin-loaded alveolar cells. Thus, it may potentially alleviate alveolar inflammation instigated by pulmonary thromboembolism [122]. Dong et al. found that breviscapine, a purified flavonoid, can downregulate IL-13 expression and further lower the expression of MCP-1 in rats with APE. Eventually, the APE-induced inflammation of lung tissue is attenuated, which promotes the autologous thrombolytic reaction [123]. Nobiletin is another flavonoid that is associated with the inhibition of phospholipase Cγ2, protein kinase C, and Akt phosphorylation in mitogen stimulated MAPK protein kinase in collagen activated human platelets. In addition, nobiletin significantly reduced intracellular calcium mobilisation and hydroxyl radical generation, ultimately leading to inhibition of platelet activation [120]. Therefore, flavonoids are expected to be novel drugs.

#### Resveratrol

Resveratrol is a nonflavonoid compound that is extracted from a type of Thuja plant (Polygonum cuspidatum) [124]. Resveratrol improves pulmonary artery endothelial cell dysfunction and alleviates CTEPH, which is associated with a reduction in oxidative stress, platelet activation, and inflammation after treatment [125]. It was discovered that resveratrol could prevent APE-induced cardiac injury. By downregulating MALAT1 promoter efficiency, resveratrol inhibits the activation of inflammasomes, as represented by increased NLRP3 expression [126]. In addition, in rat models, MCP-1 was involved in the formation of APE-induced PH, and resveratrol downregulated the expression of MCP-1 by inhibiting APE-induced p-p38MAPK activation, which contributed to the decrease in PH [127].

#### Curcumin

Curcumin is a polyphenolic compound extracted from turmeric that has anti-inflammatory and antioxidant effects. Curcumin can improve the symptom of APE rats, inhibit the expression of endothelin-1 and thromboxane B2, significantly lower MDA levels, and promote the activity of SOD. Additionally, curcumin reduces the concentration of plasma MMP-9 to improve hemodynamics and lung injury in rats with APE [128]. Curcumin decreased microRNA-21 expression by downregulating Sp1 to upregulate PTEN and to impair the NF-κB signalling pathway, thus suppressing lung injury and inflammation in APE rats [129].

#### Shuxuetong

Shuxuetong is an injection extracted from leech and earthworm and is used for the treatment of acute cerebral infarction. A recent study demonstrated that Shuxuetong injection can effectively lower mPAP and right ventricular pressure in APE animal models. It also reduces lung damage from superoxide anions by activating the antioxidant system. Shuxuetong also upregulates Bcl-2 expression and reduces Bax and Caspase expression in APE model rats. Totally, the protective effect of Shuxuetong injection in rats with APE may include antioxidant, anti-inflammatory, and anti-apoptosis [130]. In models of cerebral microvascular endothelial cells, Shuxuetong injection reduces NF-κB p65 and vascular endothelial growth factor expression, and reduces I kappa B alpha, extracellular signal-regulated protein kinase 1/2, and I kappa B kinase phosphorylation levels [131].

## Conclusions

VTE is the third most common acute cardiovascular syndrome worldwide after myocardial infarction and stroke, imposing a significant disease burden worldwide. Oxidative stress has an irreplaceable role in maintaining normal cellular physiological functions, but excess ROS lead to DNA damage and lipid peroxidation, affecting the structure and function of the cardiovascular system. Oxidative stress has an important regulatory role in the cardiovascular system, especially in vascular endothelial cells and smooth muscle cells. ROS are involved in several aspects of acute PTE pathogenesis, such as changes in shear stress blood flow, excessive activation of leukocytes, and processes involved in ischaemia-reperfusion. The absence of shear stress blood flow induces a mechanical signalling cascade response that triggers massive ROS generation, which is more pronounced during the reperfusion period with elevated oxidative stress markers. Leukocytes interact with endothelial cells and platelets to exacerbate oxidative stress-induced myocardial tissue damage in APE and act as reinforcements for new thrombi. In addition, nitric oxide is an important gas transporter in cardiovascular tissues and is inextricably linked to the overproduction of ROS. Markers such as MMP-9, along with other biomarkers are elevated in patients with APE and may be of value in the diagnosis of APE. Standard therapeutic agents such as anticoagulants have shown anti-inflammatory effects. In consideration of life-threatening haemorrhage, some medicines are summarized here. Certain western and herbal extracts have been found to reduce pulmonary artery wall inflammation, improve hemodynamics, and inhibit right ventricular injury through antioxidative stress mechanisms. As mentioned above, the process of oxidative stress plays a crucial role in APE and its induced PH. However, most of the aforementioned evidence is derived from animal experiments or in vitro experiments, and in-depth explorations of the oxidative stress mechanisms in pulmonary circulation are needed to develop more effective therapeutic strategies. Additionally, is the therapeutic effect of the antioxidants sufficient? This needs further exploration.

## Data Availability

Not applicable.

## Abbreviations

PTE: Pulmonary thromboembolism
PVR: Pulmonary vascular resistance
PAP: Pulmonary artery pressure
APE: Acute pulmonary embolism
ESC: European Society of Cardiology
LMWH: Low-molecular weight heparin
ROS: Reactive oxygen species
DNA: Deoxyribonucleic acid
PH: Pulmonary hypertension
CTEPH: Chronic thromboembolic pulmonary hypertension
IMA: Ischemia-modified albumin
AOPPs: Advanced oxidation protein product
O_2_^−^: Superoxide
H_2_O_2_: Hydrogen peroxide
NOX: Nicotinamide adenine dinucleotide phosphate oxidase
NOS: Nitric oxide synthase
XO: Xanthine oxidase
eNOS: Endothelial nitric oxide synthase
NO: Nitric oxide
SIRT2: Sirregulin-2
NF-κB: Nuclear factor κB
SOD: Superoxide dismutase
CAT: Catalase
GPx: Glutathione peroxidase
Nrf2: Nuclear erythroid-2-related factor-2
GSH: Glutathione
ONOO^−^: Peroxynitrite
CD: Cluster of differentiation
cGMP: Cyclic guanosine monophosphate
iNOS: Inducible nitric oxide synthase
mPAP: Mean pulmonary arterial pressure
PASMCs: Pulmonary artery smooth muscle cells
ATP: Adenosine triphosphate
AP-1: Activator protein-1
VTE: Venous thromboembolism
EMPs: Endothelial microparticles
sICAM-1: Soluble intercellular cell adhesion molecule-1
PAF: Platelet-activating factor
CINC-1: Cytokine-induced neutrophil chemoattractant-1
MCP-1: Monocyte chemotactic protein-1
MIP-1: Macrophage inflammatory protein
NETs: Neutrophil extracellular traps
PDMPs: Platelets and platelet-derived microparticles
HMGB1: High mobility group box 1
sVCAM-1: Soluble vascular cell adhesion molecule-1
MMPs: Matrix metalloproteinases
IL: Interleukin
ADMA: Asymmetric dimethylarginine
PRMTs: Protein arginine methyltransferases
DDAH: Dimethylarginine dimethylamine hydrolase
HSP: Heat shock protein
PaO_2_: Arterial partial pressure of oxygen
SaO_2_: Arterial oxygen saturation
Hcy: Homocysteine
COX: Cytochrome oxidase
GGT: Gamma-glutamyl transferase
NLR: Neutrophil/lymphocyte ratio
cTnI: Cardiac troponin I
NT-proBNP: N-terminal pro-brain natriuretic peptide
PAB: Prooxidant-antioxidants balance
FRAP: Ferric reducing antioxidant power
DOACs: Direct oral anticoagulants
APJ: Apelin receptor
MDA: Malondialdehyde
PAR: Protease-activated receptor
